# The first complete mitochondrial genome of Blossom-headed Parakeet *Psittacula roseata* (Psittaciformes: Psittacidae)

**DOI:** 10.1080/23802359.2020.1825131

**Published:** 2020-09-29

**Authors:** Jiansong Zhang, Chenguang Zhao, Lingling Zu, Yubao Duan

**Affiliations:** a Key Laboratory for Conserving Wildlife with Small Populations in Yunnan, Southwest Forestry University, Kunming, China; b Key Laboratory for Forest Resources Conservation and Utilization in the Southwest Mountains of China, Ministry of Education, Southwest Forestry University, Kunming, China; c Faculty of Biodiversity Conservation, Southwest Forestry University, Kunming, China

**Keywords:** *Psittacula roseata*, Blossom-headed parakeet, mitogenome, phylogeny

## Abstract

Blossom-headed Parakeet *Psittacula roseate* is listed as Near Threatened on the IUCN Red List of Threatened Species because of its habitat loss, and declining population. In this study, we first sequenced and described the complete mitochondrial genome and phylogeny of *P. roseata*. The whole genome of *P. roseata* was 16,814 bp in length, and contained 14 protein-coding genes, 22 transfer RNA genes, 2 ribosome RNA genes, and 1 non-coding control regions. The overall base composition of the mitochondrial DNA was 31.88% for A, 22.00% for T, 32.88% for C, 13.23% for G, with a GC content of 46.11%. A phylogenetic tree strongly supported that genus *Psittacula* closely related with genus *Eclectus* by highly probability.

Blossom-headed Parakeet (*Psittacula roseate*) occurs in the lowlands to c.1,500 m, inhabiting light forest, including savanna, secondary growth, forest edge, clearings and cultivated areas (Juniper and Parr 1998). *Psittacula roseata* occurs in South and South-East Asia, ranging from eastern India and Bangladesh, through Myanmar, Thailand, Laos, Cambodia, Vietnam and southern China (Yunnan, Guangxi and Guangdong provinces) (Juniper and Parr 1998). It is particularly associated with dry deciduous forest throughout south-east Asia. It is listed as Near Threatened on the basis that it is undergoing a moderately rapid decline owing to habitat loss and trapping pressure (BirdLife International 2019; IUCN. 2019). Molecular studies supported closely related with *P. roseata* and *P. cyanocephala* by highly probability based on *cytochrome-b gene* (Kundu et al. 2012). *Psittacula roseata* was the basal lineage within *Himalayapsitta* cluster, *P. cyanocephala-finschii* were a sister clade to *P. roseate* based on *cytochrome-b gene*, RAG-1 (Braun et al. 2019). The complete mitochondrial genome of *P. roseata* has not been determined and characterized until now. Therefore, the aim of this study was to first assemble and characterize the complete mitochondrial genome of *P. roseata*.

The specimen (Duan-001) was collected from Dehong Wildlife rescue center (24.37 N, 98.48E), which was located southwestern of Yunnan Province in China, and stored at the Herbarium of Southwest Forestry University. The total mitochondrial DNA was extracted from the muscle tissue. The mitogenome was sequenced using Illumina Novaseq sequencing platform (Illumina, San Diego, CA) and assembled with A5-miseq v20150522 (Coil et al. 2015) and SPAdesv3.9.0 (Bankevich et al. 2012). The complete mitochondrial genome was annotated using MITOS Web server (http://mitos2.bioinf.uni-leipzig.de/index.py) (Bernt et al. 2013). The complete mitochondrial genome of *P. roseata* was submitted to the NCBI database under the accession number MK986661. Phylogenetic tree of the relationships among 24 species were presented using maximum likelihood (ML) methods available on the CIPRES Science Gateway v3.3 (Miller et al. 2010). Bayesian inference was calculated with MrBayes3.1.2 with a general time reversible (GTR) model of DNA substitution and a gamma distribution rate variation across sites (Ronquist and Huelsenbeck 2003). Sequences of Psittaciformes (*Melopsittacus undulatus* and *Agapornis roseicollis*) obtained frofm GenBank (NC_009134 and NC_011708) were used as outgroups to root trees following Kundu et al. (2012).

The complete mitochondrial genome of *P. roseata* was found to be a circular double-stranded 16,814 bp in length. A total of 39 mitochondrial genes were identified, including 14 protein-coding genes (PCGs), 22 transfer RNA (tRNA) genes, 2 ribosomal RNA (rRNA) genes, and 1 non-coding control region (D-loop). All the 14 protein coding genes contained the same start condon ATG, except that *cox1* gene started with GTG, *nad3_0* gene started with GCC. Furthermore, eleven of the PCGs used complete TAA or incomplete T(AA)/TA(A) stop codon, and other three types were AGG[*cox1*], AGT[*nad3_1*], GAA[*nad3_0*]. The length of 22 tRNA genes ranged from 65 bp (*trnF*) to 76 bp (*trnS2*). The 12S rRNA and 16S rRNA genes were located between the *trnF* and *trnL2* genes and separated by the *trnV* gene, with a length of 976 and 1584 bp, respectively. The D-loop length was 425 bp and laied between the *trnE* and *trnF* genes.

Among these genes, *nad*6 and 8 tRNAs (*trnQ*, *trnA*, *trnN*, *trnC*, *trnY*, *trnS2*, *trnP* and *trnE*) were located on the light strand (Lstrand), while all of the remaining genes were located on the heavy strand (H-strand). The overall base composition of *P. roseata* mitogenome was 31.88% for A, 22.00% for T, 32.88% for C, and 13.23% for G, A + T content is 53.88%, which is higher than G + C content of 46.11%, similar to other Psittaciformes (Liu et al. 2019, 2019).

The reconstructed phylogenetic tree showed that *P. roseate* grouped with *P. eupatria*, *P. derbiana* and *Tanygnathus lucionensis* with strong support (100% bootstrap support value) by the analyses of protein-coding genes (Figure 1). The genus *Psittacula* clade was found to be sister to the genus *Eclectus*, which is congruent with previous studies (Kundu et al. 2012; Liu et al. 2019). The complete mitochondrial genome of *P. roseate* reported here will be useful for future population genetic studies of this species and will provide essential genome resources for the ecologically important species.

**Figure 1.**
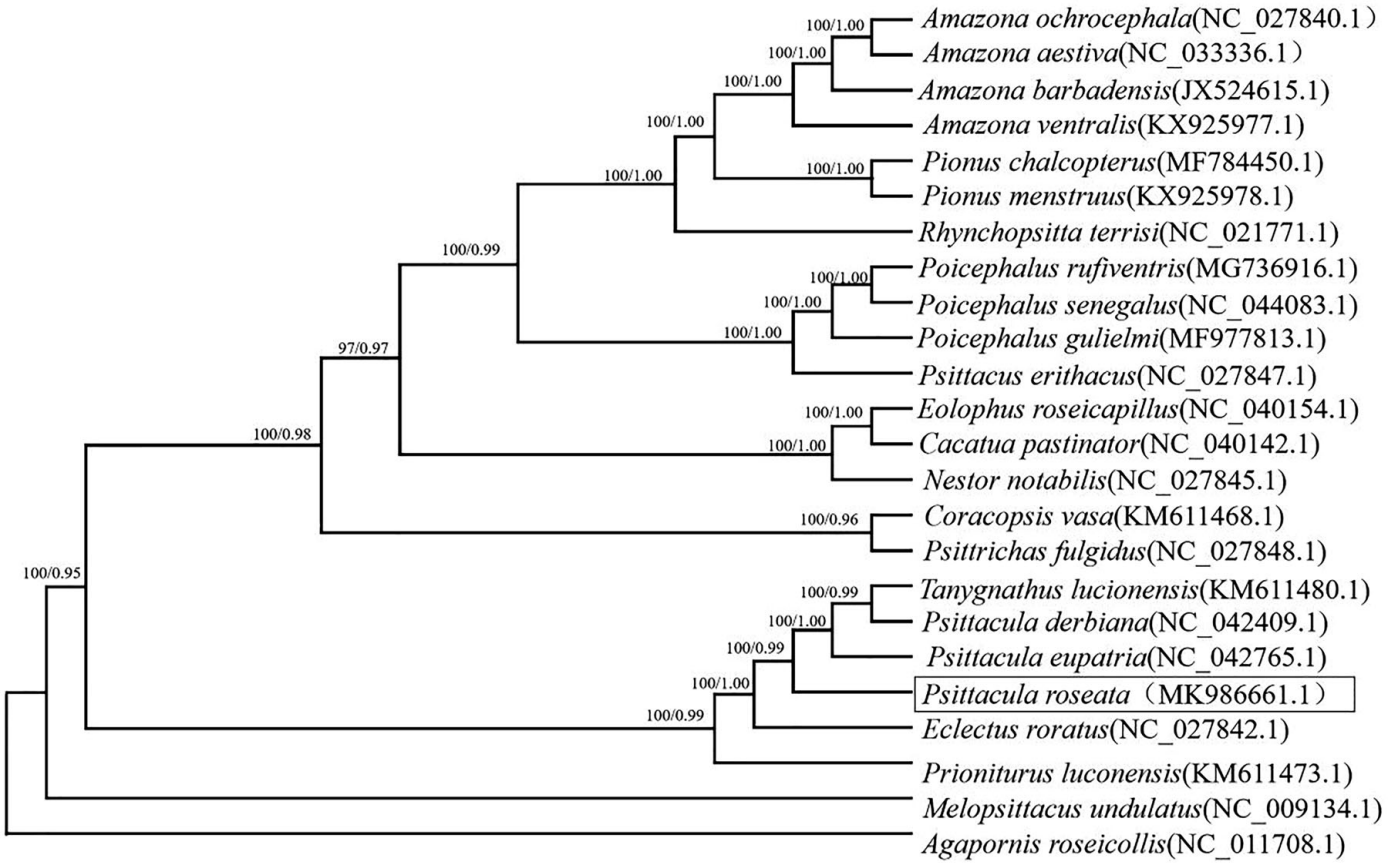
The phylogenetic tree based on combing protein-coding gene sequences of 24 speices. Numbers at node of the tree branches represent Bayesian posterior probability (left) and RAxML rapid bootstrap support (right).

## Data Availability

The data that support the findings of this study are openly available in NCBI at https://www.ncbi.nlm.nih.gov/, reference number [MK986661], or available from the corresponding author.
